# Production of aroma compounds from whey using *Wickerhamomyces pijperi*

**DOI:** 10.1186/s13568-015-0108-5

**Published:** 2015-04-16

**Authors:** Naoki Izawa, Miyuki Kudo, Yukako Nakamura, Harumi Mizukoshi, Takahiro Kitada, Toshiro Sone

**Affiliations:** Yakult Central Institute, 5-11 Izumi, Kunitachi-shi, 186-8650 Tokyo, Japan

**Keywords:** Whey, *Wickerhamomyces pijperi*, Aroma compound, Ethyl benzoate

## Abstract

The production of aroma compounds by the microbial fermentation of whey was studied. Seven strains of the yeast *Wickerhamomyces pijperi* were used for the fermentation of glucose-added whey (whey-g). Twelve aroma compounds (isobutanol, isoamyl alcohol, 2-phenylethanol, acetaldehyde, ethyl acetate, propyl acetate, isobutyl acetate, isoamyl acetate, ethyl butyrate, ethyl propionate, ethyl hexanoate and ethyl benzoate) were identified in the fermented broth using headspace gas chromatography mass spectrometry analysis. The major components were ethyl acetate (several tens to hundreds ppm), acetaldehyde (several tens ppm) and isoamyl alcohol (about 10 ppm). The strong fruity odor of ethyl benzoate (about 1 ppm) was detected in the broth of *W. pijperi* YIT 8095 and YIT 12779. The balance of aroma compounds produced was varied depending on the media used, and ethyl benzoate was only produced when using whey-g. The variation in the production of the aroma compounds over time using *W. pijperi* YIT 12779 at various culture temperatures (from 15–30°C) was also studied. From the results we propose that *W. pijperi* could be used as a novel microorganism for production of aroma compounds from whey.

## Introduction

Whey is the liquid that remains after the precipitation and removal of milk casein during cheese-making. The dairy industry generates approximately 9 kg of whey for each kilogram of cheese, and its world production is estimated to be over 10^8^ tons per year (Siso 1996; Grba et al. 2002). This whey by-product contains lactose (4.5–5% w/v), soluble proteins (0.6–0.8% w/v), lipids (0.4–0.5% w/v) and mineral salts (8–10% of dried extract). Cheese whey represents an important environmental problem because of its high organic matter content, with a BOD (Biochemical Oxygen Demand) = 30000–50000 ppm and a COD (Chemical Oxygen Demand) = 60000–80000 ppm (Siso 1996). Because of its rich nutrient content, whey has been used for the production of different chemicals such as ethanol fermentation. *Kluyveromyces marxianus*, a yeast capable of fermenting lactose to ethanol directly, has been studied in this respect. It has been used in ethanol production from crude whey (Zafar and Owais 2006), batch fermentation (Barba et al. 2001), fed-batch fermentation (Barba et al. 2001; Grba et al. 2002) and continuous fermentation (Christensen et al. 2011; Ozmihci and Kargi 2007). The production of other useful materials such as lactate, single-cell protein, enzymes and polysaccharides have also been studied using various microorganisms (Panesar and Kennedy 2012).

We are interested in studying the production of aroma compounds using whey. Aroma compounds have wide application in the food, cosmetic, chemical and pharmaceutical fields. Many aroma compounds on the market are produced via chemical synthesis or extraction from plant and animal sources. However, extraction from plants and animals is very expensive, and chemical synthesis often involves an environmentally unfriendly production process, sometimes affording undesirable racemic mixtures. Furthermore, consumers have a negative image of synthetic compounds (Vandamme and Soetaert 2002), and so (micro) biological origin aroma compounds have been the focus of much research. Many aroma compounds such as vanillin (Kaur and Chakraborty 2013; Priefert et al. 2001), 2-phenylethanol (2-PE, rose-like) (Etschmann et al. 2002), benzaldehyde (bitter almond, cherry) and 4-(*R*)-decanolide (fruity-fatty) (Krings and Berger 1998) have been observed via biotechnological production, but there are only a few studies that produce aroma compounds using whey as broth. Acetaldehyde, ethyl acetate, 1-propanol, 2-methyl-1-propanol, 2-methyl-1-butanol and 3-methyl-1-butanol were identified in whey fermented with *Kluyveromyces fragilis* (Parrondo et al. 2000). This group investigated the effects of temperature and agitation on the concentration of compounds in batch cultures, and went on to study certain compounds in continuous culture. In another study, forty aroma compounds were identified in distilled continuous whey fermentation using *K. marxianus* (Dragone et al. 2009). Whey utilization have been investigated using *Kluyveromyces* spp. and focused mainly on the ethanol fermentation (Guimarães et al. 2010). A possibility of the whey utilization would be expanded by using other microorganisms.

The aim of our study is to propose a novel method of reusing whey and adding new value through microbial fermentation, particularly with respect to the production of aroma compounds. In preliminary experiments, we noticed that a fruity odor was given off from whey fermented by the yeast *Wickerhamomyces pijperi* which were isolated from buttermilk, flower, partially decayed leaf and tree (http://www.nite.go.jp/en/nbrc/index.html). *W. pijperi* is phylogenetically closely related to *Candida solani*, and its morphologic and phylogenetic studies were examined by Imanishi et al. (2009). Only a few information is available about this yeast, to the best of our knowledge, there is only one report detecting this microorganism in the fermented liquid, Colombian Kumis (Chaves-López et al. 2012).

In this study, we investigated the *W. pijperi* fermentation of whey and analyzed the profiles of the aroma compounds produced when using different yeast strains, media and culture temperatures.

## Materials and methods

### Microorganisms and culture conditions

Seven strains of *W. pijperi* were used in this study. *W. pijperi* strain YIT (Yakult central Institute, Tokyo) 8095 (NBRC (Biological Resource Center, NITE) 1290), 12778 (NBRC 1791), 12779 (NBRC 1887), 12780 (NBRC 102058), 12781 (NBRC 102059), 12782 (NBRC 102060) and 12783 (NBRC 102061). Whey used in this study was the supernatant of 3% skimmed milk fermented using *Streptococcus thermophilus*, containing about 11 g/L lactose, 4 g/L galactose and 4 g/L lactate (no glucose present), 80 ng/L protein and had a pH of 4.0.

Twenty microliters of a 20% glycerol stock of *W. pijperi* was inoculated into 2 mL of YM (Yeast Mold) medium (Becton, Dickinson and company, Sparks, MD, USA) and cells were grown overnight at 30°C with 160 rpm reciprocal shaking (pre-preculture). Unless stated, all cultures in this study were carried out this way. For the screening of the strains, 20 μL of the pre-preculture broth was inoculated into 2 mL of whey with 10 g/L of glucose (whey-g), as *W. pijperi* cannot utilize lactose and galactose (Kurtzman and Fell 1998). Cells were further cultured overnight (preculture), then 50 μL of preculture broth was inoculated into 5 mL of whey-g in a test tube and cultured for 24 h.

For the comparison of media, 20 μL of pre-preculture was inoculated into 2 mL each of whey-g, YM medium (Becton, Dickinson and company), YPD (Yeast Extract Peptone Dextrose) medium (1% yeast extract, 2% peptone and 2% glucose) and 10% skim milk (Becton, Dickinson and company) and cultured overnight. Then, 50 μL of each culture broth was inoculated into 5 mL of the same media respectively, and cultured for 24 h.

For time-course analysis, 1 mL of whey-g preculture broth was inoculated into 100 mL of whey-g in a 200 mL Erlenmeyer flask and cultured at 15°C, 20°C, 25°C, 30°C and 35°C with 160 rpm rotary shaking. The culture broth was sampled at 0, 8, 24, 32, 48 and 56 h. All the samples were frozen at −30°C until use. Each datum point represents the mean of values from duplicate experiments.

### Headspace gas chromatography mass spectrometry (HS-GC-MS)

An HS-GC-MS and an HS-GC- flame ionization detection (FID) protocols were modified from Dragone et al. (2009) and Saerens et al. (2008a) An HS-GC-MS system was used to identify the aroma compounds. This system consists of a 7000C GC/MS triple quadrupole mass spectrometer coupled to a GC 7890B (Agilent technologies, Palo Alto, CA, USA) and multi-purpose sampler MPS2-xt (Gerstel, Mühlheim/Ruhr, Germany). The samples were centrifuged at 8,000 rpm for 5 min and 2 mL of supernatant were collected in 20 mL glass tubes. Samples were heated for 15 min at 50°C with gentle shaking in the HS autosampler. One mL of sample was delivered into InertCap PureWAX column (30 m × 0.25 mm i.d., 0.25 μm, GL sciences, Tokyo, Japan). The flow rate of the carrier gas (He) was 1.0 mL/min, the injector temperature was 250°C and the transfer line temperature was 230°C. A split injection with a ratio of 1:10 was used. The oven temperature was held at 40°C for 5 min and then programmed to increase to 250°C at 10°C/min with a final 5 min hold at 250°C. The mass spectrometer was operated as follows: ionization voltage, 70 eV; ion source, EI mode; ion source temperature, 230°C. The mass spectrometer scanned from 34 to 400 (m/z) in 300 ms. The identification was performed by comparing the obtained mass spectra to the National Institute of Standards and Technology (NIST) 2011 mass spectral library.

### HS-GC-FID

An HS-GC-FID system was used to quantify the aroma compounds. The samples were prepared in the same way as for HS-GC-MS. The system consisted of 7697A and GC7890 apparatus (Agilent technologies) with an FID detector. Samples were heated for 15 min at 50°C with gentle shaking in the head space autosampler. Five mL of sample was delivered to an InertCap PureWAX column (30 m × 0.25 mm i.d., 0.25 μm, GL sciences). The flow rate of the carrier gas (He) was 3.0 mL/min. The injector temperature was 250°C, and the loop and transfer line temperatures were 100°C and 115°C, respectively. A split injection with a ratio of 1:20 was used. The oven temperature program was the same as for the HS-GC-MS analysis. The compounds were detected using an FID detector at a temperature of 250°C. Each identified aroma compounds was purchased and used for standard. Quantification was carried out by comparing with the peak area of 50 ppm (200 ppm for ethyl acetate) of each aroma compound.

### Other analysis

Cell growth was measured at 600 nm using a Cary 60 UV-Vis spectrometer (Agilent technologies). Lactose, glucose, galactose, lactate and ethanol concentrations were quantified using a high performance liquid chromatography (HPLC) system. An HPLC protocol was modified from Chohnan et al. (2011). The system consisted of an alliance e2695 separation module (Waters corporation, Milford, MA, USA) equipped with a 2414 RI detector (Waters corporation). Ten micro liters of supernatant of culture broth was injected into a SUGAR SH1011 column (8.0 mm i.d. ×300 mm, Showa Denko, Tokyo, Japan) with 5 mM H_2_SO_4_ as eluent at 0.5 mL/min. The column and the detector temperatures were at 30°C and 37°C, respectively.

## Results

### Aroma compounds production of *W. pijperi*

Eleven aroma compounds were identified in whey-g fermented using *W. pijperi* (Table 1). Almost all of these compounds have a fruity aroma impression. The composition of aroma compounds varied depending on the strain of yeast used. High concentrations of acetaldehyde and ethyl acetate were observed, followed by isobutanol and isoamyl alcohol, and then isoamyl acetate in all culture broths. High production of ethyl acetate was observed in *W. pijperi* YIT 12779, 12780 and 12783. Ethyl benzoate was detected in the culture broth of *W. pijperi* YIT 8095 and 12779, which gave off an especially strong fruity odor. The odor of these two culture broths were recreated artificially using commercially available reagents at the detected concentration, and found that ethyl benzoate is important constituents of the fruity odor.

**Table 1:** **Aroma compounds produced by** ***W. pijperi*** **strains grown in whey-g**

**Compound (ppm)**	**8095**	**12778**	**12779**	**12780**	**12781**	**12782**	**12783**	**Aroma impression**
**Alcohols**								
Isobutanol	8.15	7.25	7.41	9.58	6.94	5.33	9.37	Solvent^a^
Isoamyl alcohol	10.07	10.85	10.79	11.67	8.24	6.78	11.91	Alcoholic, banana^a^
**Aldehyde**								
Acetaldehyde	17.99	14.77	13.01	20.93	23.82	32.61	15.36	Fruity, solvent^b^
**Acetate esters**								
Ethyl acetate	42.72	80.17	198.38	174.55	63.89	75.91	159.20	Fruity, solvent^a^
Propyl acetate	0.00	0.13	0.29	1.22	0.00	0.00	0.24	Pineapple^b^
Isobutyl acetate	0.00	0.00	0.11	0.08	0.00	0.07	0.11	Fruity aroma^a^
Isoamyl acetate	0.04	0.17	0.30	0.21	0.28	0.08	0.24	Banana^a^
**Ethyl esters**								
Ethyl propionate	0.00	0.25	0.34	0.27	0.00	0.00	0.25	Fruity, rum^b^
Ethyl butyrate	0.60	0.14	0.61	0.13	0.00	0.00	0.14	Apple, banana^b^
Ethyl hexanoate	0.05	0.04	0.05	0.04	0.00	0.00	0.05	Apple, fruity^a^
Ethyl benzoate	1.14	0.00	1.24	0.00	0.00	0.00	0.00	fruity^c^

a: Pires et al. (2014).

b: Liu et al. (2004).

c: Niu et al. (2011).

### Influence of culture media in aroma compounds composition

The effects of the culture media on the aroma compounds composition were investigated. Whey-g, skim milk, YM and YPD were fermented using *W. pijperi* YIT 8095 and 12779 as they produced a strong odor in the initial whey-g experiments.

The composition of aroma compounds produced varied depending on the media. High production of ethyl benzoate, which has a characteristic fruity odor, was observed in whey-g with both strains. Isobutanol and isoamyl alcohol were highly produced in skim milk and YPD, respectively. 2-PE was only produced in YM and YPD (Table 2).

**Table 2:** **Difference in aroma compounds composition in different media**

**Compound (ppm)**	**12779**				**8095**			
	**Whey-g**	**Skim milk**	**YM**	**YPD**	**Whey-g**	**Skim milk**	**YM**	**YPD**
**Alcohols**								
Isobutanol	15.17	33.80	17.04	27.53	14.79	25.97	15.86	13.34
Isoamyl alcohol	7.53	39.61	98.31	235.70	17.07	28.10	70.17	242.14
2-PE	0.00	0.00	27.81	28.49	0.00	0.00	14.73	30.60
**Aldehyde**								
Acetaldehyde	1.66	9.90	18.03	46.30	11.02	9.08	20.95	39.90
**Acetate esters**								
Ethyl acetate	305.73	7.18	102.18	234.76	69.05	3.52	28.43	291.34
Propyl acetate	0.98	0.00	0.00	0.67	0.20	0.00	0.00	0.69
Isobutyl acetate	1.25	0.07	0.10	0.00	0.08	0.00	0.00	0.00
Isoamyl acetate	5.07	0.24	1.56	3.09	0.58	0.00	0.38	2.07
**Ethyl esters**								
Ethyl propionate	1.01	0.18	1.17	2.12	0.57	0.00	0.86	2.37
Ethyl butyrate	0.57	0.56	1.08	0.80	0.55	0.27	1.00	3.25
Ethyl hexanoate	0.04	0.35	0.05	0.12	0.08	0.32	0.15	0.24
Ethyl benzoate	2.78	0.18	0.11	0.00	2.69	0.14	0.09	0.10

### Influence of fermentation temperature on *W. pijperi* YIT 12779 growth and carbon source consumption

**Figure 1:**
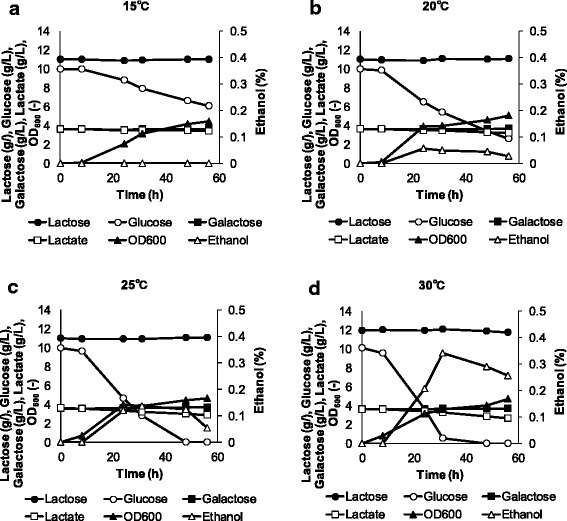
**Time courses of growth, sugar and lactate consumption and ethanol production.** *W. pijperi* YIT 12779 was cultured at **(a)** 15°C, **(b)** 20°C, **(c)** 25°C and **(d)** 30°C in the whey-g. Growth (*black triangles*), glucose (*white circles*), galactose (*black squares*), lactose (*black circles*), lactate (*white squares*) and ethanol (*white triangles*).

*W. pijperi* YIT 12779 was chosen for further research because of the distinctive fruity odor of its culture broth. The time courses for growth and carbon source consumption at various temperatures in whey-g are given in Figure 1 (this strain cannot grow at 35°C). Glucose was consumed first, followed by lactate at 25°C and 30°C, and the fastest glucose consumption was observed at 30°C. Compared with other temperatures, slow growth was observed at 15°C (Figure 1). Lactose and galactose were not consumed during the fermentation. Higher ethanol production was observed at higher temperatures, reaching 0.3% after 32 h at 30°C.

### Influence of fermentation temperature on *W. pijperi* production of aroma compounds

**Figure 2:**
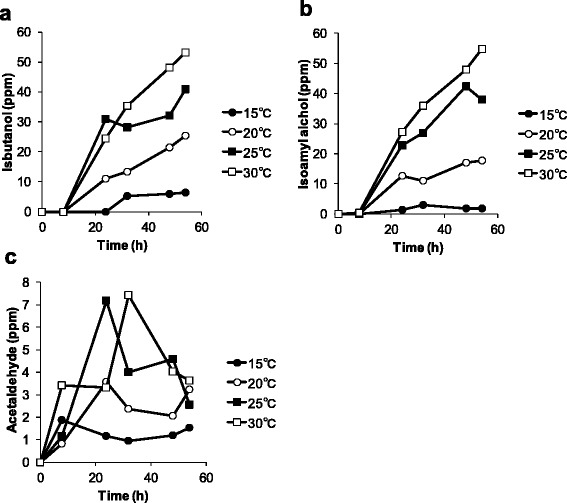
**Time courses of alcohols and acetaldehyde concentrations at each temperature.** *W. pijperi* YIT 12779 was cultured in the whey-g. The concentrations of **(a)** isobutanol, **(b)** isoamyl alcohol and **(c)** acetaldehyde. *Black circles*: 15°C, *white circles*: 20°C, *black squares*: 25°C and *white squares*: 30°C.

**Figure 3:**
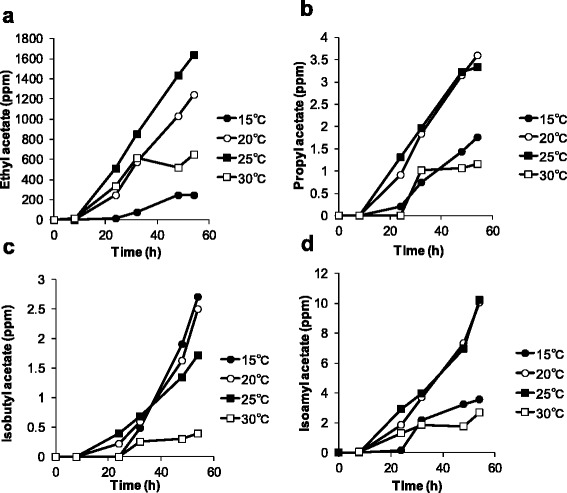
**Time courses of acetate esters concentrations at each temperature.** *W. pijperi* YIT 12779 was cultured in the whey-g. The concentrations of **(a)** ethyl acetate, **(b)** propyl acetate, **(c)** isobutyl acetate and **(d)** isoamyl acetate. *Black circles*: 15°C, *white circles*: 20°C, *black squares*: 25°C and *white squares*: 30°C.

**Figure 4:**
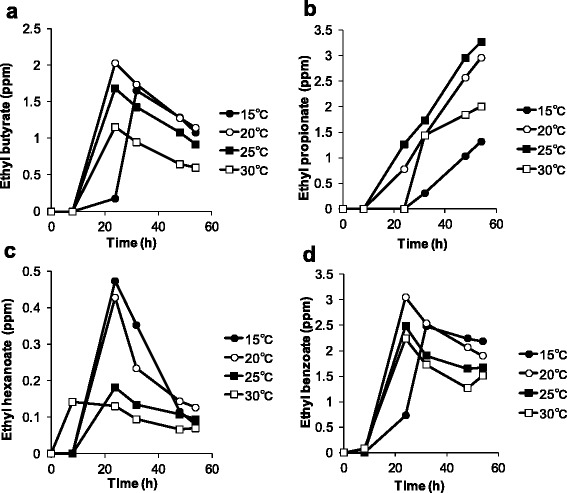
**Time courses of ethyl esters concentrations at each temperature.** *W. pijperi* YIT 12779 was cultured in the whey-g. The concentrations of **(a)** ethyl butyrate, **(b)** ethyl propionate, **(c)** ethyl hexanoate and **(d)** ethyl benzoate. *Black circles*: 15°C, *white circles*: 20°C, *black squares*: 25°C and *white squares*: 30°C.

Figures 2, 3, 4 show the effect of temperature on the production of aroma compounds. *W. pijperi* YIT 12779 was grown in whey-g at 15, 20, 25 and 30°C. Eleven aroma compounds were grouped as follows: alcohols (isobutanol and isoamyl alcohol) and aldehydes (acetaldehyde) (Figure 2); acetate esters (ethyl acetate, propyl acetate, isobutyl acetate and isoamyl acetate) (Figure 3); ethyl esters (ethyl propionate, ethyl butyrate, ethyl hexanoate and ethyl benzoate) (Figure 4). There were profile differences within the same group, for example, 3 acetate esters showed high production at 20°C and 25°C, but isobutyl acetate was also significantly produced at 15°C (Figure 3). In addition, the concentration of ethyl propionate kept increasing over the timeframe of the experiment, but levels of the other 3 ethyl esters decreased during the stationary phase (Figure 4).

## Discussion

In this study, we found that whey-g fermentation using *W. pijperi* produces aroma compounds (Table 1). Twelve aroma compounds were identified in whey-g, skim milk, YM and YPD media (Table 2). Several tens ppm of isobutanol and isoamyl alcohol were detected in the whey fermented by *K. fragilis* (Parrondo et al. 2000). Additionally, several hundreds ppm of these compounds and ppb order of minor compounds were detected in the distilled *K. marxianus* fermented whey spirits (Dragone et al. 2009). So the level of isobutanol and isoamyl alcohol concentrations of *W. pijperi* fermented whey-g were low and the other minor aroma compounds were the same level as the *Kluyveromyces* spp. fermented whey. All of the aroma compounds had already been reported in *Saccharomyces* and *Kluyveromyces* fermentation, except for ethyl benzoate (Pires et al. 2014; Medeiros et al. 2000; Molina et al. 2007). It is novel that ethyl benzoate was produced from whey and *W. pijperi* possessed the ability to make it. The strong fruity odor was identified in *W. pijperi* YIT 8095 and 12779 fermented broth, these strains were collected from butter milk (Kurtzman et al. 2008) and partially decayed leaf, respectively (Mitaka and Banno 1979). Imanishi et al. (2009) showed that *W. pijperi* YIT 8095, 12778, 12779 and 12783 can be classified as neighbors by phylogenetic analysis based on three different nucleotide sequences (D1/D2 domain of 26S rDNA, the actin gene *ACT1* and the elongation factor 2 gene *FE2*). *W. pijperi* YIT 8095 and YIT 12779 are especially closely related according to D1/D2, and these 2 strains produced ethyl benzoate with levels up to 3 ppm (Tables 1 and 2). It is interesting that the ethyl benzoate strongly contributes to the fruity odor, considering that the absolute concentration of ethyl benzoate was very low compared with ethyl acetate, acetaldehyde, isoamyl alcohol and isobutanol. The phenotype that produces the strong fruity odor from YIT 8095 and YIT 12779 is extremely specific because YIT 12778 and YIT 12783 do not produce a fruity odor, even though these four strains are phylogenetically related.

The composition of the aroma compounds produced varied depending on the culture media (Table 2). Saerens et al. (2008a) noted that the precursor level present in the media can affect ester production while researching *Saccharomyces cerevisiae*. Likewise, a different composition of media could also affect the *W. pijperi* aroma compound production. Isoamyl alcohol was highly produced in YPD, and no production of 2-PE was observed in whey-g and skim milk. Considering that leucine and phenylalanine are precursors of isoamyl alcohol and 2-PE, respectively, this demonstrates that differences in the amino acid composition of the media affects the composition of the aroma compounds produced (Dickinson et al. 1997; Etschmann et al. 2002).

It is interesting that ethyl benzoate can be produced from whey-g but not from skim milk. Lactic acid bacteria, such as *S. thermophilus*, that are involved in the fermentation of skim milk have cell-envelope proteinase and peptide transport systems that use exogenous protein (Savijoki et al. 2006). Extracellular proteinases can also release peptides from proteins in the culture medium (Yamamoto et al. 1994). This goes some way to explaining how *S. thermophilus* can convert milk protein into peptides, which can be utilized by *W. pijperi* to produce ethyl benzoate.

In the time-course experiments, glucose was consumed first, then lactate consumption began after the glucose levels had depleted when *W. pijperi* YIT 12779 was studied at temperatures up to 30°C (Figure 1). We confirmed *W. pijperi* YIT 8095 also did not use lactose and galactose experimentally. Referred to the NCYC (National Collection of Yeast Cultures) website (http://www.ncyc.co.uk/), *W. pijperi* do not use these sugars generally. The next challenge will be lactose and galactose utilization by *W. pijperi* in whey. This would be a great advantage environmentally, and gene manipulation is likely to be the best strategy to accomplish this. Although it has not yet been reported for *W. pijperi*, there are some reports demonstrating similar transformations in assortative *Pichia pastoris* (Morton and Potter 2000; O’Callaghan et al. 2002; Yuan et al. 2008).

Time-course studies of the production of aroma compounds were shown to be affected by temperature (Figure 2, 3, 4). Saerens et al. (2008a; 2008b) also reported that the production profile of esters by *S. cerevisiae* was affected differently by temperature, depending on the type of ester. High concentration of alcohols (ethanol, isobutanol and isoamyl alcohol) were obtained at high temperature, and levels of isobutanol and isoamyl alcohol tend to increase as time went on (Figures 1 and 2). The differences in the production profiles may be due to the differences in the biosynthetic pathways. Ethanol is produced at the end of the glycolytic pathway, but isoamyl alcohol and isobutanol are produced during leucine and valine synthesis, respectively (Dickinson et al. 1997; Matsuda et al. 2012).

Acetaldehyde was detected at higher levels at 25°C and 30°C, and the concentration decreased during the stationary phase (Figure 2c). Acetaldehyde is synthesized from pyruvate and metabolized to ethanol during ethanol fermentation. The profile for acetaldehyde may appear different to the other compounds as synthesis and consumption occur simultaneously. The observed decrease may also be due to evaporation during fermentation and sample preparation as the boiling point is very low (20.2°C).

Ester formation has been well studied in many organisms (Park et al. 2009), and the alcohol o-acyltransferases (ATF) are one class of enzymes involved in this process. In *S. cerevisiae*, acetate esters are produced from acetyl-CoA and alcohol using alcohol acetyltransferases, and ethyl esters are produced by a condensation reaction between an acyl-CoA unit and ethanol using acyl-CoA/ethanol o-acyltransferases. Although these enzymes have not been specifically observed in *W. pijperi*, it may work in a similar way. It is interesting that there was a difference in production between isoamyl acetate and isobutyl acetate, which are formed by the combination of branched fatty acids and acetic acid (Figure 3). It has been shown that ATFs have preferences for substrates (Rodriguez et al. 2014), so this difference may be due to the ATF in *W. pijperi* favoring isobutanol over isoamyl alcohol.

Ethyl propionate was shown to increase over time, but the levels of the 3 other ethyl esters decreased in the stationary phase (after 20 hours) (Figure 4). It is unlikely that these compounds could be metabolized to other substances. Although propionate is supplied directly via isoleucine metabolism, there is no direct amino acid direct supplier for hexanoate, benzoate and butyrate (KEGG: Kyoto Encyclopedia of Genes and Genomes, http://www.genome.jp/kegg/). It is thought that the different precursor supplementation pathways may cause the observed differences in the profiles, and that evaporation exceeded synthesis for the 3 esters that showed a decrease in the stationary phase.

We found that ethyl benzoate was important components of the fruity odor by sniffing artificially prepared samples. It is very important aroma compound used for fragrance, flavor and solvents. However, only a few papers discuss ethyl benzoate being found in fermentation products (Chen and Xu 2010; Zea et al. 2001) as it is mainly detected in fruit (Bartley and Schwede 1989; Thuaytong and Anprung 2011). To the best of our knowledge, fermentative production of ethyl benzoate has not yet been reported.

The biotechnological production of aroma compounds is very attractive for the reasons outlined in the introduction. Furthermore, as it is independent from agriculture and any potential disruptions to production, it can be carried out on an industrial scale using engineered pathways to produce what are regarded as natural products (Krings and Berger 1998). The results of this study will help to develop a novel bio production of aroma compounds. Further studies will be necessary to increase aroma compound production by investigating detailed fermentation conditions and developing genetically engineered yeast strains.

## Abbreviations

YIT: Yakult Central Institute, Tokyo
NBRC: Biological Resource Center, NITE
YM: Yeast mold
YPD: Yeast extract peptone dextrose
HS-GC-MS: Headspace gas chromatography mass spectrometry
HS-GC-FID: Headspace gas chromatography flame ionization detection
HPLC: High performance liquid chromatography
NCYC: National collection of yeast cultures
ATF: Alcohol o-acyltransferases
KEGG: Kyoto encyclopedia of genes and genomes
